# Response to the letter on “Comments to ‘PRevention of INCisional hernia after liver transplantation (PRINC trial): study protocol for a randomized controlled trial’ by Janusz Strzelczyk”

**DOI:** 10.1186/s13063-020-04244-y

**Published:** 2020-04-14

**Authors:** T. Auer, D. Kniepeiss, P. Schemmer

**Affiliations:** 1 General, Visceral and Transplant Surgery, Medical University of Graz, Auenbruggerplatz 29, 8036 Graz, Austria; 2 Transplant Center Graz, Graz, Austria

To the editor,

We are very grateful to Dr. Janusz Strzelczyk for showing interest in the study protocol “PRevention of INCisional hernia after liver transplantation (PRINC trial): study protocol for a randomized controlled trial” [1] and for communicating his concerns about choosing an absorbable mesh for incisional hernia prevention after liver transplantation (LT). Indeed, to the best of our knowledge, this study is the first to investigate incisional hernia prevention with a synthetic mesh placed with the usual onlay technique during abdominal wall closure in LT.

Prophylactic onlay mesh placement has been very successfully investigated in non-immunosuppressed patients, and results of the PRIMA study, a randomized controlled trial, were published recently [2]. Furthermore, synthetic meshes have been used uneventfully (especially without increased surgical side infections) in immunosuppressed patients with existing incisional hernia [3]. However, our study combines, for the first time, the prophylactic onlay approach with a resorbable mesh after LT. This study is important since (a), especially early after LT, immunosuppression is high and wound healing is compromised and (b) non-resorbable meshes are prone to get superinfected or to induce seroma with associated problems [4, 5].

Moreover, during the early post-operative course after LT, factors such as malnutrition due to end-stage liver disease and large abdominal surgery further increase the risk for inflammation, infection, and less effective collagen synthesis, resulting in a higher incidence of incisional hernia. The articles cited by Dr. Strzelczyk are not focused on the early post-transplant period. They only compare hernia following LT during the long-term follow-up with hepatopancreatic surgery [3] or focus on the late post-operative period after LT [6]. These studies have not shown an increased incidence of incisional hernia recurrence that is due to long-term immunosuppression. The clear working hypothesis is that prophylactic placement of a slow resorbable mesh during LT should protect against the most frequently occurring hernias during the early post-operative course [7].

Even with non-resorbable onlay placed meshes, recurrent hernias are reported in up to 32% of midline incisions within 5 years [8]. Most importantly, the surgical skill of abdominal wall repair is the only independent factor for the recurrence of an incisional hernia, according to the “expertise in abdominal wall surgery matters” trial, in which experts had only 12% recurrent hernias, which was almost 60% lower than in the non-expert group [9].

Results concerning the use of biological and bio-absorbable meshes were presented recently [10]. The data analysis demonstrated that both biological and bio-absorbable meshes could not be recommended for the use of complex hernia repair. In case of biological meshes increased inflammatory activity and inacceptable high recurrence rates occurred. Bio-absorbable meshes were not investigated sufficiently for a routine recommended use. The successful use of P4HB meshes was shown by a multicenter study group [11, 12] that performed a multicenter prospective study including contaminated ventral hernia repairs. The recurrence rate was 9% after 4 years in the US study group and after 2 years in the European study group. Both results were presented at international conferences (of the Americas Hernia Society and the European Hernia Society (EHS)). Our own observation study of 46 patients with complex and mostly contaminated ventral hernia showed a 4-year recurrence rate of 6% (presented at the EHS meeting, 2019).

Thus, Phasix® has been chosen for the PRINC trial because of its unique properties, namely low inflammatory response, high resistance to bacterial colonization, and high mechanical strength [13, 14].

In conclusion, the PRINC study protocol is based on both local and international data and therefore represents an appropriate attempt to prevent the most prevalent early occurrence of post-transplant hernias.

## Data Availability

Not applicable.

## Abbreviations

EHS: European Hernia Society
LT: Liver transplantation
PRINC: PRevention of INCisional hernia after liver transplantation
